# Contribution of growth hormone secretagogue receptor (GHSR) signaling in the ventral tegmental area (VTA) to the regulation of social motivation in male mice

**DOI:** 10.1038/s41398-021-01350-6

**Published:** 2021-04-20

**Authors:** Su-Bin Park, Samantha King, David MacDonald, Anne Wilson, Harry MacKay, Barbara Woodside, Alfonso Abizaid

**Affiliations:** Department of Neuroscience, Carleton University, Ottawa, ON Canada

**Keywords:** Physiology, Neuroscience

## Abstract

Most psychiatric disorders are characterized by deficits in the ability to interact socially with others. Ghrelin, a hormone normally associated with the regulation of glucose utilization and appetite, is also implicated in the modulation of motivated behaviors including those associated with food and sex rewards. Here we hypothesized that deficits in ghrelin receptor (growth hormone secretagogue receptor; GHSR) signaling are also associated with deficits in social motivation in male mice. To test this hypothesis, we compared social motivation in male mice lacking GHSR or mice treated with the GHSR antagonist JMV2959 with that of WT or vehicle-treated mice. GHSR signaling in dopamine cells of the ventral tegmental area (VTA) has been implicated in the control of sexual behavior, thus we further hypothesized that GHSR signaling in the VTA is important for social motivation. Thus, we conducted studies where we delivered JMV2959 to block GHSR in the VTA of mice, and studies where we rescued the expression of GHSR in the VTA of GHSR knockout (KO) mice. Mice lacking GHSR or injected with JMV2959 peripherally for 3 consecutive days displayed lower social motivation as reflected by a longer latency to approach a novel conspecific and shorter interaction time compared to WT or vehicle-treated controls. Furthermore, intra-VTA infusion of JMV2959 resulted in longer latencies to approach a novel conspecific, whereas GHSR KO mice with partial rescue of the GHSR showed decreased latencies to begin a novel social interaction. Together, these data suggest that GHSR in the VTA facilitate social approach in male mice, and GHSR-signaling deficits within the VTA result in reduced motivation to interact socially.

Social interaction is an essential facet of the behavioral repertoire of animals and can be influenced by environmental perturbations. Not surprisingly, the pathologies associated with chronic stress typically involve disturbances in social behaviors and often lead to social isolation and anxiety^1–4^. In laboratory animals, chronic social defeat results in increased caloric intake, weight gain, and adiposity, as well as increased social anxiety and depressive-like behaviors^2–5^. Chronic social defeat also results in persistent increases in the release of plasma ghrelin^5,6^, a hormone associated with increased caloric intake and carbohydrate utilization^7^. Both of these functions are important for meeting the energetic demands associated with chronic stress, and their absence may lead to vulnerability to stress-induced pathology^8^. For example, following chronic social defeat, growth hormone secretagogue receptor (GHSR) knockout (KO) mice show decreased place preference for palatable foods and spend less time investigating a novel conspecific^9^. Similar effects are found when stress-induced ghrelin secretion is reduced by administration of β−1 adrenergic receptor antagonists^10^. Furthermore, rescue of the GHSR in tyrosine hydroxylase positive cells restores both food preference and reduces social anxiety in stressed GHSR KO mice^9^. Together, these data suggest that GHSR signaling is important not only for the metabolic consequences of chronic stress exposure, but also for its effects on social interaction.

Ghrelin has also been implicated in the modulation of social behavior in experimental paradigms that do not involve stress. For instance, male GHSR KO mice or mice treated with GHSR antagonists show longer latencies to approach receptive females and have lower preference scores for females than WT mice or mice that are treated with saline^11,12^. Similar findings have been observed in rats that have a point mutation in the GHSR gene leading to a truncated protein that results in abnormal GHSR signaling^13^. Male rats from this strain exhibit normal sex behaviors although they show a longer latency to approach a novel receptive female and have lower locomotor activity in anticipation of receptive females^13^. While these data have been interpreted as demonstrating lower motivation to engage in sexual behavior, they may reflect a general deficit in the motivation to engage socially. Finally, mice receiving chronic infusions of the ghrelin receptor antagonist JMV2959 for 4 weeks show longer latencies to approach unfamiliar mice^14^, highlighting the importance of GHSR signaling in modulating social interactions, as increased latencies could suggest decreased motivation to interact with a conspecific.

Social motivation is mediated in part through the activation of dopamine cells in the ventral tegmental area (VTA)^15^. These cells receive inputs from hypothalamic regions important for the integration of sensory and hormonal signals, and project to a number of forebrain regions including the nucleus accumbens (NAc), prefrontal cortex (PFC), amygdala and hippocampus^16^. The release of dopamine into these brain regions is associated with reward seeking behaviors including those that facilitate social interactions^16,17^. Interestingly, direct administration of ghrelin into the VTA excites dopaminergic neurons leading to increased dopamine turnover in the NAc^18,19^, which might be expected to result in changes in social behavior. Dopamine cells within the VTA also contain receptors for, and respond to, peptides associated with social motivation including oxytocin, galanin, and neurotensin, and the neurons that produce these peptides are located in hypothalamic regions that also express GHSR, providing another route through which ghrelin may influence social behavior^15,20–25^. Together these data provide evidence to suggest that GHSR signaling could modulate social behavior in the absence of chronic stress. To investigate this possibility, we examined the effects of genetic or pharmacological manipulation of GHSR signaling on social motivation. Our results show that pharmacological or genetic deletion of the GHSR leads to deficits in social motivation, and that rescue of GHSR signaling in the VTA restores social motivation in GHSR KO mice.

## General methods

### Animals

All experimental and stimulus mice used in this study were male and weighed between 25–30 g at the onset of the experiments. Mice used in the pharmacological experiments were C57BL/J6 mice purchased from Jackson Laboratory (Bar Harbor ME, USA). Stimulus mice were WT C57BL/J6 mice obtained from our transgenic breeding colony and were paired-housed with one other mouse before being used in our study. We also used two different strains of transgenic mice that did not express the GHSR (GHSR KO). The first strain of GHSR KO mice were provided by Dr. Tamas Horvath at Yale University and were engineered with a mutation in the promoter region that prevented GHSR expression and instead expressed the *LacZ* reporter (GHSR^*Lacz/Lacz*^). These mice originate from breeding pairs developed by Regeneron Pharmaceuticals Inc. (Tarrytown, NY, USA) and were previously characterized^26^. The second strain of transgenic mice lacking GHSR were provided by Dr. Jeffrey Zigman at UT Southwestern, and contain the insertion of a transcriptional blocking cassette on the putative promoter region of the GHSR gene preventing transcription. This transcriptional blocking cassette is flanked by *LoxP* sites and can therefore be removed through CRE-mediated recombination. This model has been previously characterized^27^ and we refer to it as GHSR^*LoxP/LoxP*^. Both transgenic models were backcrossed onto a C57BL/6 strain and bred in our facilities. Mice were genotyped using primer sequences as described in previous papers^18,27^. All mice were single housed in standard plastic mouse cages (27 × 21 × 14 cm) for a period of 10 days before any experimental manipulations and in a temperature-controlled (22 ± 1 °C) and humidity-controlled (50 ± 5%) environment on a 12 h light–dark cycle (lights on at 08:00 h) with access to chow (2.9 kcal/g, with 70% of calories derived from carbohydrates) and water at ad libitum. Mice were assigned randomly to their respective experimental groups. All experimental procedures were approved by the Carleton University Animal Care Committee and followed the guidelines of the Canadian Council on Animal Care.

### Drug treatments

In some experiments, we injected JMV2959 (Millipore), a GHSR receptor antagonist to examine the effects of blocking GHSR activity globally or locally in the VTA. The GHSR receptor antagonist JMV2959 was chosen in this study as it is a small non-peptidergic compound that can be delivered peripherally and centrally to prevent the feeding effects of ghrelin^28,29^.

#### Systemic ghrelin administration

To block GHSR globally we injected mice intraperitoneally (i.p.) with either JMV2959 (3 mg/kg) or a similar volume of vehicle (isotonic saline) once a day for 3 consecutive days. Mice were injected between 9:00 and 10:00 AM every morning and were tested 30 min after the last i.p. injection. This peripheral dose was chosen because it is in the midrange of those used systemically in previous studies and has been used successfully to attenuate the reinforcing properties of several drug types including alcohol, opioids, and stimulants^30–33^.

#### Intra-VTA ghrelin delivery

Infusions of JMV into the VTA were given at a dose of 6 μg/day at a rate of 0.11 μl/h for a period of 7 days as was the vehicle solution (isotonic saline). The dose of JMV2959 delivered into the VTA with osmotic minipumps was chosen from studies using acute intracerebral infusions to oppose the effects of ghrelin on psychostimulant effects in rats^34^ and sex behaviors in mice^12^.

Mice were anesthetized with isoflurane mixed with oxygen (4%), injected subcutaneously with analgesic meloxicam (2 mg/kg, Metacam^®^), and secured onto a mouse stereotaxic apparatus (Kopf Instruments, Tujunga, CA). Following aseptic surgical protocols, the skull was exteriorized and a small hole was made at the appropriate site to allow for insertion of an L-shaped 30-gauge stainless steel cannula (Plastics One Model 330OP/DW/Spc) aimed at the VTA using coordinates from the Paxinos and Franklin mouse brain atlas^35^ (AP-2.92, ML+/−0.7, DV-4.5). The cannula was connected to an osmotic minipump (Alzet model 1004) via a PE-20 polyethylene catheter (Fisher Scientific) measuring at least 2.5 cm to allow the animal to have full range of motion. The cannula was secured with a small screw that, along with the base of the cannula, was covered with dental cement. The minipump was then inserted into a space created in the intrascapular region. The skin was the sutured and covered with topical antibiotic (Polysporin^®^). Mice were allowed to recover from surgery for a period of 10 days before undergoing the behavioral tests described below.

Upon conclusion of behavioral testing, mice were injected with a lethal dose of Dorminal (1 mg/kg i.p.; CDMV, Quebec, Canada) and perfused transcardially with 0.9% saline, followed by 4% paraformaldehyde in 0.1 M phosphate buffer. Brains were then extracted and post-fixed in 4% paraformaldehyde for 24 h, followed by submersion in a 30% sucrose solution (w/v) prior to sectioning. 40 µm coronal sections containing the VTA (Figs. 55–63 in ref. ^35^) were sliced at −21 °C on a Thermo Fisher Scientific cryostat. Sections were then viewed under a light microscope to verify the location of cannula. All mice with evidence of misplaced cannula were assigned to vehicle–sham or JMV2959 sham groups and served as anatomical controls for their respective treatment conditions.

### Rescue of GHSR expression in the VTA of GHSR KO mice

To rescue GHSR in the VTA we used an approach similar to that of Skov et al.^36^. We gave bilateral microinfusions of either an adeno-associated vector (AAV) vector expressing the green fluorescent protein (GFP; AAV9.hSyn.eGFP.WPRE.bGH; Addgene; 1 × 10¹³ vg/mL) or AAV-CRE recombinase fused to GFP (AAV9.hSyn.HI.eGFP-Cre.WPRE.SV40; Addgene; 7 × 10¹² vg/mL) into the VTA of GHSR^*LoxP/LoxP*^ mice, and WT littermates. The infusions were performed using similar surgical protocols as cannula implantation, except that a 33-gauge stainless steel guide cannula (Plastics One model 330OP/DW/Spc) connected to a microsyringe was inserted into the VTA to deliver the assigned virus at a flow rate of 0.5 μl in 400 s. The concentration of the virus was at a ratio of 1:2 of the original stock. This procedure was then repeated to target the contralateral VTA. Mice underwent behavioral testing three weeks following stereotaxic infusion of the AAV into the VTA. At the end of the study, mice were decapitated, and their brains were extracted and flash frozen in ice-cold 100% ethanol (EtoH) and stored at −80 °C. These brains were processed for RTqPCR to determine relative expression of GHSR in the VTA and a control region above the VTA (the Edinger–Westphal nuclei) in GHSR^*LoxP/LoxP*^ and WT mice with and without the CRE-mediated rescue. The Edinger–Westphal nucleus was chosen as a control area because it lies dorsal to the VTA and also expresses GHSR^20^. 500 µm-thick coronal sections of the midbrain containing the VTA and the Edinger–Westphal nucleus sections were collected ranging across the anterior posterior plane from −2.92 and −3.88 mm from Bregma. These sections were chosen based on a mouse brain atlas^35^ Bilateral 1 mm punches of the VTA and Edinger–Westphal nucleus were collected from the frozen sections using a modification of the method described by Palkovits^37^. These punches were then processed for RTqPCR as described below.

### Reverse transcription quantitative polymerase chain reaction (RTqPCR)

Total RNA was isolated from homogenized brain punches using TRIzol (Life Technologies) and precipitated using linear acrylamide. Total RNA was dissolved in 20 μl of DEPC-treated, nuclease-free, deionized water. RNA concentration and purity were determined by measuring the absorbance at 260 nm and the ratio of absorbance at 260 and 280 nm (A260/280) on a Nanodrop Lite spectrophotometer (Thermo Fisher Scientific). RNA quality was verified by gel electrophoresis on a 1% agarose gel stained with ethidium bromide (0.4 μg/mL, Sigma Aldrich), and imaged on an Invitrogen E-Gel Imager with UV Base (Thermo Fisher Scientific). cDNA was generated by reverse transcription using the iScript Reverse Transcription Kit (Bio-Rad Laboratories Inc., Hercules, CA) according to the manufacturer’s directions. The RT qPCR reaction was conducted on all cDNA samples to determine relative expression of target genes using the 2^−ΔΔCt^ method. 5 µl of cDNA sample were added to each well of a 96-well PCR plate, followed by 2 µl of working concentration of primer solution, 3 µl of DEPC water, and 10 µl of SsoAdvanced^TM^ SYBR^®^ Green Super Mix with Fluorescein (Bio-Rad Laboratories Inc., Hercules, CA). Samples were run in duplicate alongside non-template controls (NTCs) and positive controls. The plates were run on a CFX Real-time PCR detection system (Bio-Rad) and data collected through CFX Manager 3.0 software.

All primers were supplied by Eurofins Genomics (Louisville, KY). Primer annealing temperatures were verified experimentally by running reactions at a range of annealing and extension temperatures, and a standard curve was constructed to determine both reaction efficiency and the optimal cDNA concentration. Reaction efficiency for all primers was between 90% and 110% (see Table 1 for primer sequences). Wild type mice treated with the control virus served as the reference group.

**Table 1:** Primers used to determine GHSR expression in the VTA and the Edinger–Westphal Nucleus.

Gene	Forward sequence	Reverse sequence
Mouse β-Actin	GAACCCTAAGGCCAACCGTG	GGTACGACCAGAGGCATACA
Mouse GHSR	CTCAGGGACCAGAACCACAAAC	ACAAAGGACACCAGGTTGCAG

### Behavioral tests

#### Social interaction test

The social interaction test was conducted following the method of Tsuda and Ogawa^38^. Briefly, mice were habituated for 48 h to a chamber that contained a perforated cylinder (3.5″(d) by 7″(h); Plastics Ottawa), a covered nest box, nestlet and a wooden block. On the test day, all enrichment except the perforated cylinder was removed from the chamber and the mouse acclimatized to this condition for 30 min. A novel mouse of the same strain was then placed into the centrally located perforated cylinder and social activity was video recorded for 10 min. Social motivation was measured operationally as latency to approach the novel mouse and the frequency of sniffing the stranger mouse during the test. We also measured social vigilance which was operationally defined as number of stretches towards, and corner observations of, the novel mouse recorded during the test^38–40^.

#### Open field test

The open field test was conducted as described by Selberhene and Wooten^41^. Mice were placed individually in a corner of the open field box (50 × 50 × 50 cm) and allowed to freely explore for 5 min. Behavior during this time was video recorded and the time spent (seconds) in the periphery of the box was used as a measure of anxiety-like behavior. At the end of the 5-min open field test, mice were immediately exposed to the novelty suppressed feeding test (see below).

#### Novelty suppressed feeding test

A modified version of the original novelty suppressed feeding test developed by Britton and Britton^42^ was used in which mice were tested under ad libitum conditions. A similar test was recently described by Lockie et al.^43^ to demonstrate that peripheral ghrelin increases food motivation in an anxiogenic environment. Mice were given access to a palatable snack (either Pilsbury™ cookie dough or a 60% high fat diet pellets (Harlan)) for 48 h and this was removed from the home cage 24 h before the novelty suppressed feeding test. At all times the mice consumed all of the palatable food provided, showing a preference for this diet. After the open field test (see above), each experimental mouse was briefly removed from the open field and a dish containing the palatable snack to which they had been previously exposed was placed in the middle of the open field. The mice were then placed back into a corner of the open field and were videotaped for 5 min to measure the latency to approach the food and the number of food approaches during the test. The quantity of palatable food consumed was measured at the end of the test.

To ensure that the order of tests did not influence behavioral performance, tests were administered 7 days apart in a counterbalanced order. Thus, half of the mice in each group underwent the social interaction test on test day 1 first, and the open field test immediately followed by the novelty suppressed feeding test first on test day 2. The order of test presentation was reversed for the remaining mice. Mice were tested under ad lib conditions in all experiments. The behavioral scoring of videos was done blind to the conditions by two independent raters with a correlation of 0.93 in their score ratings.

### Statistical analyses

Student’s *t*-tests were used to compare behavior and gene expression between genotypes (GHSR-WT vs. GHSR-KO) and drug treatments (saline vs. JMV2959). A similar *t*-test was conducted to analyze the effects of intra-VTA infusions of vehicle vs. JMV2959. Data obtained after GHSR rescue in the VTA were analyzed using one-way analyses of variance (ANOVA). Where appropriate, post-hoc tests were performed using Tukey’s honest significant difference (HSD). All statistical analyses were performed using Prism software. A critical value for significance was set at an alpha level = 0.05. All data are presented as mean ± SEM.

## Results

### GHSR signaling is critical for the full display of social and food motivated behaviors

**Fig. 1:**
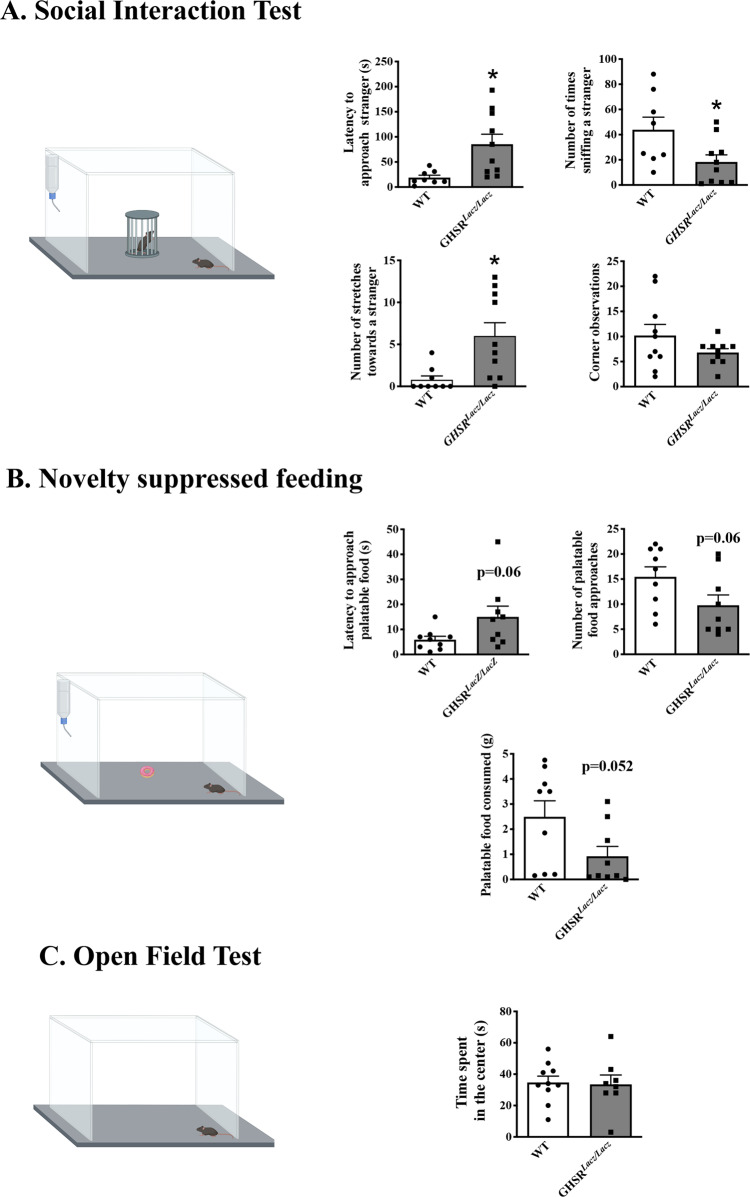
Behavioral responses in GHSR KO mice of the GHSR^*Lacz/Lacz*^ strain. As shown in Panel **A**, GHSR^*Lacz/Lacz*^ mice (*n* = 10) showed longer latencies to approach a stranger and a lower frequency of investigation than their WT littermates (*n* = 8) (*p* < 0.05). In contrast, GHSR^*Lacz/Lacz*^ mice showed more corner observations, a behavior associated with social vigilance (*p* < 0.05). As shown in Panel **B**, GHSR^*Lacz/Lacz*^ mice show similar latency to approach a palatable snack, approach the snack with the same frequency, and consumed similar amounts of the palatable snack as WT mice (see Panel **B**). Finally, GHSR^*Lacz/Lacz*^ mice did not differ from WT mice on behavior in the open field test (see Panel **C**). *p < 0.05.

Previous work from our lab and that of others demonstrated that mice and rats lacking a functional GHSR, or treated with GHSR receptor antagonists, show deficits in behaviors associated with the motivation to obtain food or sex rewards^9,11–14,44–46^. As shown in Fig. 1A, results from our first experiment showed that GHSR^*Lacz/Lacz*^ mice displayed a longer latency to approach an unfamiliar conspecific (*t*(16) = 2.9, *p* < 0.01, *η*^2^ = 0.34), and investigated these novel mice less as reflected in a lower frequency of sniffing the novel con-specific (*t*(16) = 2.34, *p* < 0.05, *η*^2^ = 0.255). In contrast, GHSR^*Lacz/Lacz*^ showed more behaviors that have been described as social vigilance^39,40^. For instance, these GHSR null mice showed significantly more stretching behaviors from the corner of the testing box towards the novel mouse without actually approaching the mouse or entering the interaction zone where they could be near the novel mouse (*t*(16) = 2.34, *p* < 0.05, *η*^2^ = 0.35).

Figure 1B depicts results from the novelty suppressed feeding test. As seen in this figure, GHSR^*Lacz/Lacz*^ mice showed no significant deficits in food motivation in these mice. In this test, latency to approach a preferred palatable snack of GHSR^*Lacz/Lacz*^ mice was not different from that of WT littermates (*t*(16) = 2.01, *p* = 0.06, *η*^2^ = 0.20; see Fig. 1B). GHSR^*Lacz/Lacz*^ mice and WT mice approached the food with comparable frequency (*t*(16) = 1.97, *p* = 0.06, *η*^2^ = 0.19; see Fig. 1B), and to eat similar amounts of the palatable diet ((*t*(16) = 2.1, *p* = 0.052, *η*^2^ = 0.21; see Fig. 1B).

No group differences in behavior were observed in the open field test suggesting that GHSR^*Lacz/Lacz*^ mice do not have deficits in general exploratory behavior that would account for the deficits in social or food motivation in these same animals (*p* > 0.05; see Fig. 1C).

**Fig. 2:**
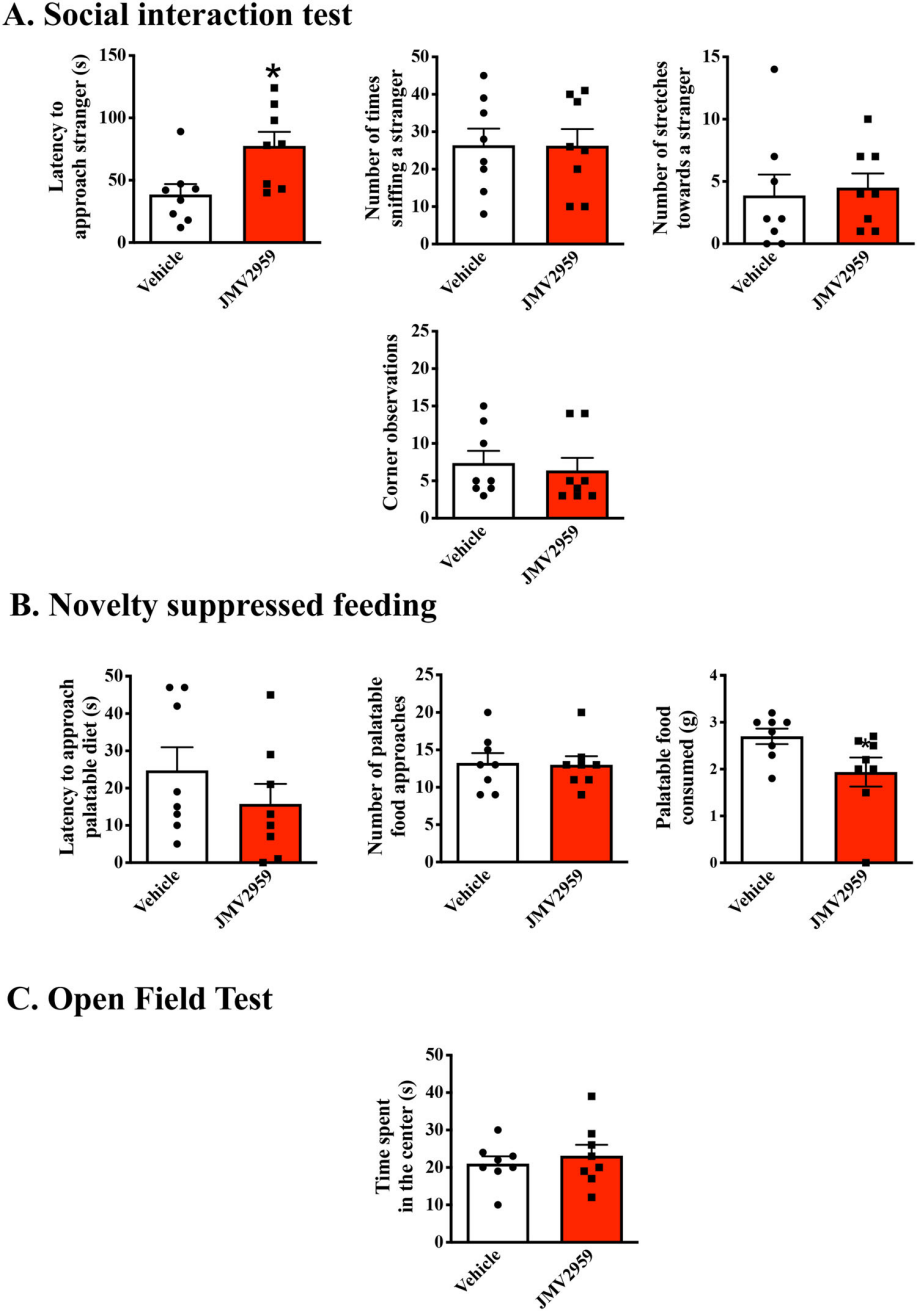
Behavioral responses of mice receiving peripheral injections of the GHSR antagonist JMV2959 (3 mg/kg) (*n* = 8) or saline (*n* = 8). As seen in panel **A**, peripheral injections of JMV2959 resulted in increased latencies to approach a novel mouse (*p* < 0.05). Treatment with the GHSR antagonist did not affect the latency to approach a palatable snack compared to control mice but it did decrease the amount of food consumed by these mice once they approached the food (*p* < 0.05); see Panel **B**). Finally, peripheral JMV2959 treatment did not affect behavior in the open field test (see Panel **C**). *p < 0.05.

Like GHSR^*Lacz/Lacz*^ mice, C57BL/J6 mice injected peripherally with the GHSR antagonist JMV2959 showed a significantly longer latency to approach a novel con-specific than saline treated mice (*t*(14) = 2.75, *p* < 0.01, *η*^2^ = 0.35; Fig. 2A), although, once they approached they showed a similar frequency of sniffing behavior (*p* > 0.05; see Fig. 2A). No other differences were observed in social behaviors between JMV2959 and vehicle-treated mice (*p* > 0.05). Unlike GHSR^*Lacz/Lacz*^ mice, mice injected i.p with JMV2959 showed similar latencies to approach a palatable snack and similar number of palatable food approaches in the novel context as vehicle-treated mice (*p* > 0.05, see Fig. 2B). Treatment with JMV2959 however, did significantly decrease the amount of palatable food consumed by mice compared to vehicle treated mice (*t*(14) = 2.17, *p* < 0.05, *η*^2^ = 0.25 Fig. 2G). As with GHSR^*Lacz/Lacz*^ mice, JMV2959 treatment did not influence behavior in the open field test (*p* > 0.05; see Fig. 2C).

### GHSR signaling in the VTA is critical for the display of social motivation

**Fig. 3:**
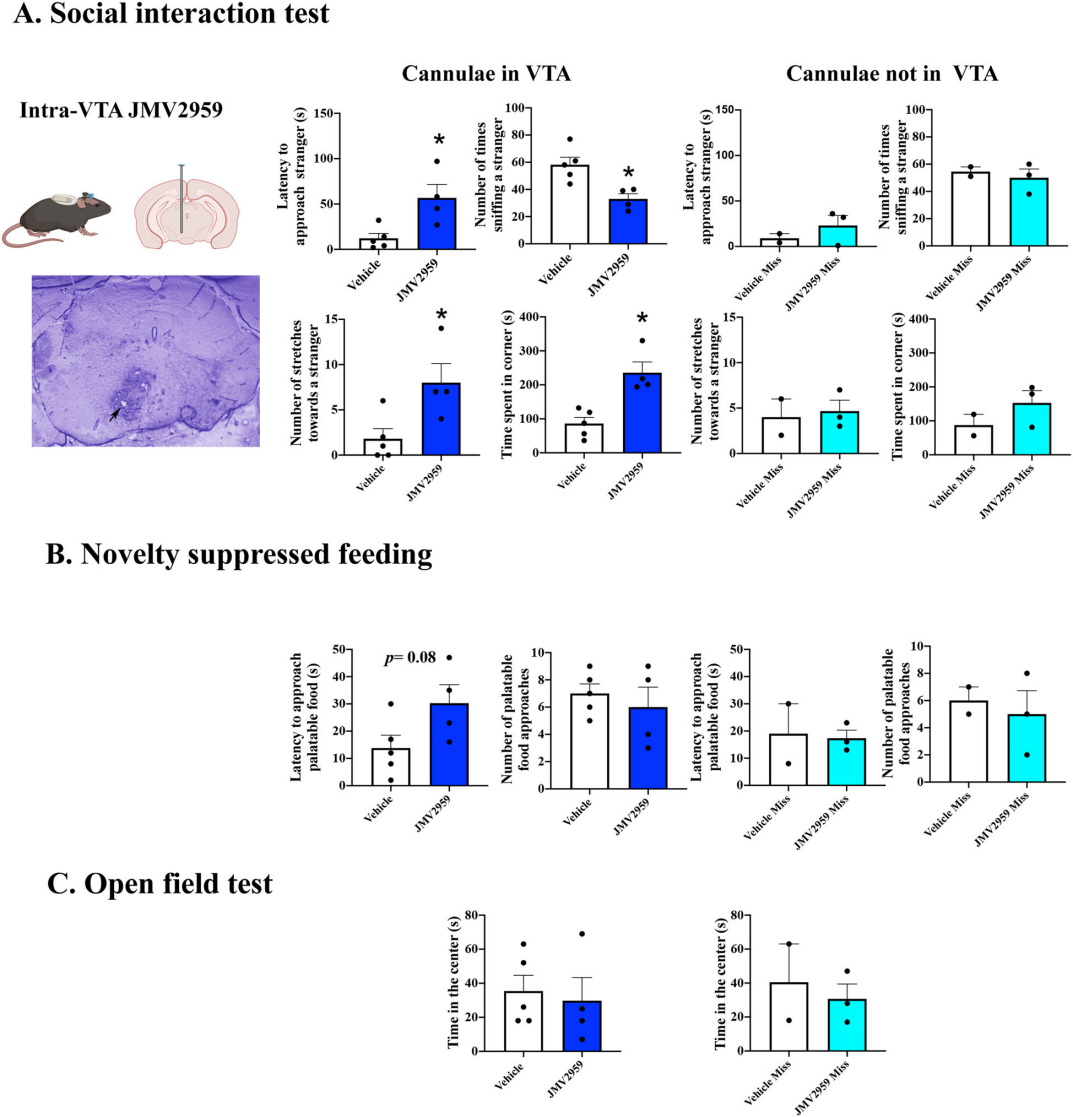
Behavioral responses of mice receiving chronic unilateral intra-VTA infusions of the GHSR antagonist JMV2959 (6 μg/day at a rate of 0.11 μl/h)(*n* = 4 with cannula in the VTA) or saline (*n* = 5). Intra-VTA treatment with JMV2959 decreased the latency to approach and the frequency of investigation of a stranger con-specific (see Panel **A**; *p* < 0.05). This treatment also resulted in increased vigilance as reflected in an increase in the number of stretches and in the amount of time spent observing the stranger from the corner of the testing arena (see Panel **A**; *p* < 0.05). Intra VTA treatment with JMV2959 was not effective in increasing the latency to approach a palatable snack (see Panel **B**). Finally, intra-VTA JMV2959 treatment did not affect behavior in the open field test (see Panel **C**). No significant differences between groups were observed in mice where the cannula placements were outside of the VTA. *p < 0.05.

Ghrelin receptors are found in a number of brain regions associated with feeding and affective states^20^. Of these, GHSR signaling in the VTA can influence motivation for food and sex^9,12,13,47^. To investigate whether GHSR signaling in the VTA influenced social interactions between same sex conspecifics, we delivered JMV2959 or vehicle chronically into the VTA of C57BL/J6 mice. Of the original *N* = 7 mice per group in only five vehicle-treated and four JMV2959-treated mice was the cannula correctly placed in the VTA. As shown in Fig. 3A, Analysis of data from these mice, showed that JMV2959 delivery into the VTA produced social motivation deficits similar to those observed in GHSR^*Lacz/Lacz*^ mice. Thus, mice infused with JMV2959 into the VTA showed a longer latency to approach novel conspecifics (*t*(7) = 3.096, *p* = 0.017, *η*^2^ = 0.57), spent less time investigating these novel mice (*t*(7) = 3.521, *p* = 0.009, *η*^2^ = 0.63), and showed more corner observations (*t*(7) = 4.31, *p* = 0.003, *η*^2^ = 0.72), and more stretches towards the stranger mouse but without entering the interaction zone (*t*(7) = 2.75, *p* = 0.02, *η*^2^ = 0.52).

In contrast to the behavior of GHSR^*Lacz/Lacz*^ mice and as shown in Fig. 3B, mice treated with JMV2959 into the VTA showed no differences in the latency to approach palatable food in the NSFT compared to controls (*t*(7) = 2.04, *p* = 0.08, *η*^2^ = 0.37; Fig. 3B). Subsequent to this, JMV2959-treated mice approached the palatable food the same number of times as controls (*p* > 0.05). Furthermore, blocking the GHSR in the VTA did not affect behavior in the open field (*p* > 0.05; see Fig. 3C). Animals treated with JMV2959, but with a cannulae that missed the VTA were not different from vehicle-treated mice with missed cannulae on any of these measures (*p* > 0.05; Fig. 3A, B and C right side panels).

**Fig. 4:**
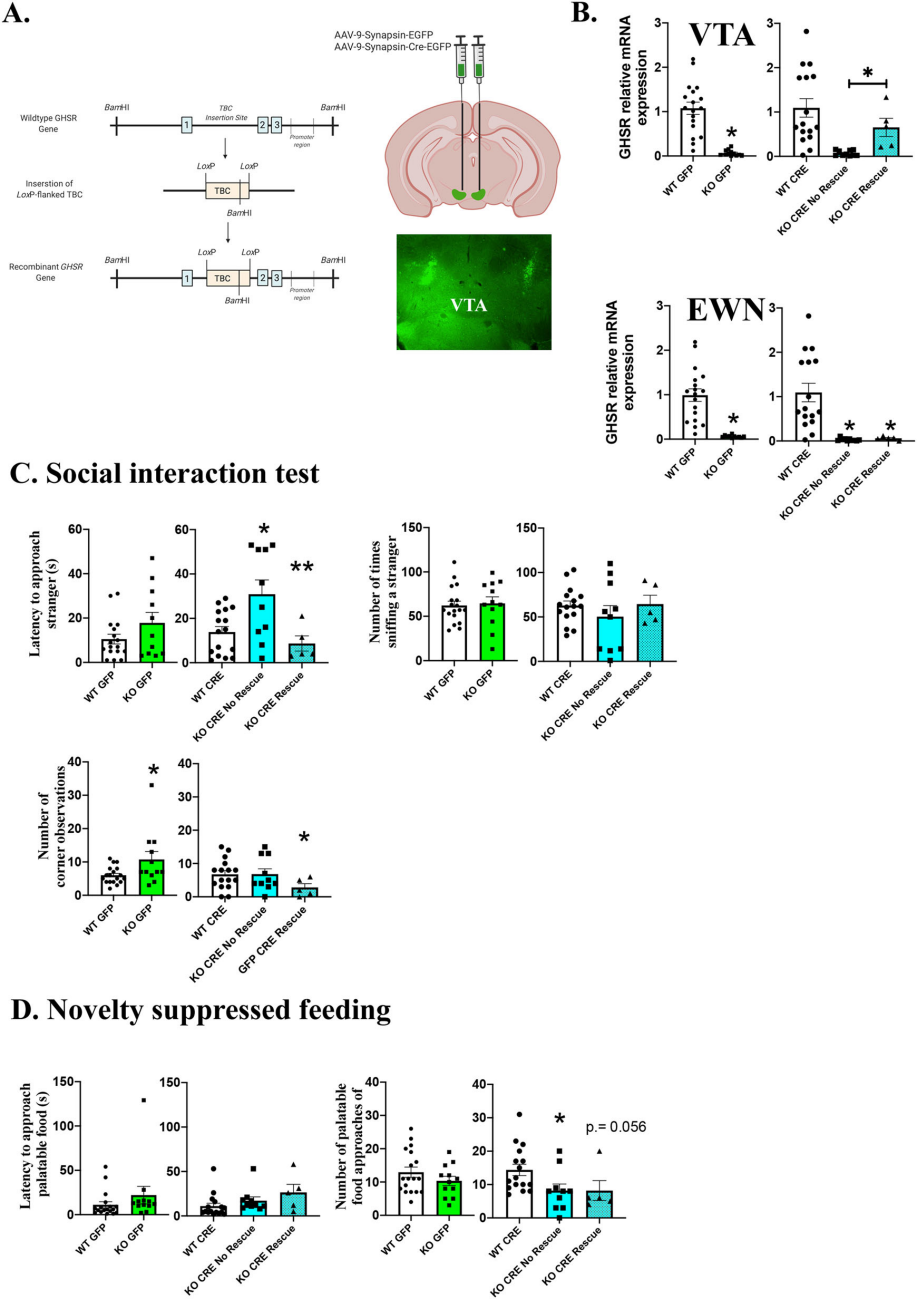
Behavioral responses in GHSR KO mice of the GHSR^*LoxP/LoxP*^ strain after CRE-mediated rescue of expression. As shown in Panel **A**, the GHSR^*LoxP/LoxP*^ strain contains a Loxp-flanked transcriptional blocking cassette (TBC) on the putative promoter region of the GHSR gene preventing GHSR expression. In the presence of CRE-recombinase, this TBC can be removed to restore expression at the site of recombination. Using Viral mediated CRE-recombination (see Panel **A**), we were able to rescue GHSR expression in a subset of mice (*n* = 5) to about 60% of control WT mice given a GFP expressing control virus (*n* = 18; see Panel **B**). The rescue was specific to the VTA since no changes in GHSR expression were observed in the EWN of the same mice (see Panel **B**). Analyses on the behavioral responses of these mice compared to those of unsuccessful GHSR rescue showed that this partial rescue was sufficient to reduce the latency to approach a conspecific compared to that of GHSR^*LoxP/LoxP*^ mice that received the control GFP (*n* = 12) virus or GHSR^*LoxP/LoxP*^ where the rescue was unsuccessful (*n* = 10; *p* < 0.05; see Panel **C**). Partial rescue of GHSR in the VTA was not sufficient to reduce the latency to approach a palatable food snack nor the frequency to approach the snack in the novel environment (*p* > 0.05; see Panel **D**). *Significantly different from control WT mice, *p* < 0.05; **significantly different from mice with unsuccessful rescue of GHSR mRNA expression, *p* < 0.05.

To further investigate the role of GHSR signaling in the VTA in modulating social motivation, we conducted a study in which we restored GHSR expression in the VTA of GHSR^*LoxP/LoxP*^ using an AVV vector that expresses CRE-recombinase under the transcriptional control of the synapsin promoter so that CRE was expressed only in neurons at the infusion site (pENN.AAV.hSyn.HI.eGFP-Cre.WPRE.SV40; see Fig. 4A). Control mice were infused with a similar virus containing the reporter gene only (pAAV.hSyn.eGFP.WPRE.bGH). Three weeks after the infusion of the virus, mice were tested in the same behavioral tasks as in the previous three experiments. Brain punches containing the VTA were processed for RTqPCR to determine the extent of GHSR rescue in GHSR^*LoxP/LoxP*^ mice infused with the Cre-expressing vector. GHSR^*LoxP/LoxP*^ mice treated with the control GFP vector showed little if any GHSR expression in the VTA. Mice infused with the Cre-expressing virus however, showed increased GHSR mRNA expression in the VTA that was close to 30% of WT control mice. This increase in GHSR expression was statistically significant from GHSR^*LoxP/LoxP*^ mice treated with the control GFP-expressing virus (*t*(25) = 2.064, *p* = 0.049, *η*^2^ = 0.15; see Fig. S1). The infusions were selective to the VTA as analyses of GHSR mRNA in the Edinger–Westphal nucleus of GHSR^*LoxP/LoxP*^ infused with the CRE-expressing virus showed little GHSR expression after following transduction (see Fig. S1). This nucleus lies dorsal to the VTA and has been shown to express GHSR^20^.

A closer observation of the data in Fig. S1 showed considerable variability in the efficacy of the viral infection such that only 5 of the 15 GHSR^*LoxP/LoxP*^ mice infused with the CRE-expressing virus showed increases in VTA GHSR mRNA expression that was above 20% of WT expression. When analyzed on their own, these five animals showed 65% of VTA GHSR mRNA expression of that from control WT mice (see Fig. 4B). The rest showed little or no increase in VTA GHSR mRNA expression (7% of WT GHSR expression in the VTA) and were not different from GHSR^*LoxP/LoxP*^ mice given the control GFP expressing virus (6% of WT expression). This subset of GHSR^*LoxP/LoxP*^ mice did not show increased GHSR mRNA expression in the Edinger–Westphal nucleus (see Fig. 4B). We therefore decided to analyze the data from those mice where VTA GHSR mRNA expression was higher than 20% of controls as a separate group (KO CRE rescue), from those where the rescue failed to significantly increase GHSR expression (KO CRE No Rescue) and compared their level of GHSR expression with that of WT littermates also infused with the CRE virus as well as mice in the WT GFP and KO GFP groups. A one-way ANOVA followed by Tukey’s post hoc tests determined KO CRE rescued mice showed a significant increase in GHSR mRNA expression compared to KO CRE no rescue and KO GFP mice (*F*(4,55) = 11.27, *p* < 0.0001, *η*^2^ = 0.255; see Fig. 4B). There was no similar change in GHSR expression in the Edinger–Westphal nucleus of KO CRE rescue mice, again suggesting that the rescue was specific to the VTA.

Figure 4C shows behavioral responses in the social interaction task. As seen in this figure, the viral rescue of GHSR expression in GHSR^*LoxP/LoxP*^ mice resulted in lower latencies to approach a novel conspecific compared to mice in the KO CRE No-Rescue group and were comparable to the latencies observed by control WT mice infused with the CRE-expressing virus (*F*(4,55) = 4.52, *p* = 0.003, *η*^2^ = 0.10, Tukeys HSD, *p* < 0.05, *p* > 0.05, respectively). In contrast, KO No Rescue mice, showed longer latencies to approach unfamiliar conspecifics than WT CRE mice (*p* < 0.05) and were comparable to GHSR^*LoxP/LoxP*^ infused with the GFP virus. There was no significant difference across groups, however, in the amount of time mice investigated the unfamiliar mouse once they made an approach (see Fig. 4C). Finally, a one-way ANOVA (*F*(4,56) = 2.75, *p* = 0.03, *η*^2^ = 0.16) followed by post hoc Tukey tests determined that KO Rescue mice showed, on average, fewer corner observations than control GFP expressing GHSR^*LoxP/LoxP*^ and KO No Rescue mice (see Fig. 4C).

In contrast to social approach, analyses of behavior in the NSFT failed to show significant differences between the groups in the latency to approach food (*F*(4,56) = 1.12, *p* = 0.36; see Fig. 4D). Although GHSR^*LoxP/LoxP*^ mice infused with the GFP expressing vector showed fewer food approaches than WT mice infused with the same vector, this effect only approached significance (*F*(4,56) = 2.23, *p* = 0.07, *η*^2^ = 0.13; see Fig. 4D). GHSR rescue in the VTA was not effective in increasing the number of food approaches in these animals (see Fig. 4D).

Mice in the GHSR rescue group spent less time in the center portion of the open field compared to WT mice or to KO No Rescue mice, but this effect was not statistically significant (*p* > 0.05; data not shown).

## Discussion

The most notable finding from our experiments is that the GHSR, and specifically GHSR in the VTA, is important for behaviors linked to social motivation. Our results show that two different strains of GHSR null mice took longer to approach an unfamiliar conspecific and at least one of these strains (the GHSR^*LacZ/LacZ*^ strain) investigated this unfamiliar mouse less than WT littermates. Similarly, both peripheral and intra-VTA administration of JMV2959 increased latencies to approach and reduced social investigation of a novel mouse, relative to vehicle-treated mice. These data support the hypothesis that the GHSR in the VTA plays an important role in social motivation. This idea is further supported by recent data showing deficits in sex motivation in rats and mice lacking the GHSR, or in mice or rats treated with GHSR antagonists peripherally or directly into the VTA^12–14^. In rats in particular, blocking GHSR signaling decreases sex anticipation without affecting sexual performance, suggesting that GHSR signaling in the VTA is selective for behaviors that are motivational in nature^13^.

The current finding that unstressed GHSR^*LoxP/LoxP*^ mice, once they approach the novel conspecific, spend similar amounts of time investigating it relative to WT mice is consistent with previous studies examining the effects of chronic social stress on social behavior in GHSR^*LoxP/LoxP*^ mice^5,9^. Unfortunately, latency to approach the novel conspecific, a major measure of social motivation in the current study, was not reported in these earlier studies. The notion that GHSR signaling in the VTA has its major effect on initiating rather than maintaining contact with the stranger mouse is consistent with the idea that GHSR signaling, and particularly GHSR signaling in the VTA is important for initiating social exploration^12–14,48^.

To avoid confounding the anxiogenic effects of placing an animal in an unfamiliar context, in the current studies we used a modified social interaction test described by Tsuda and Ogawa^38^ in which mice are habituated to the environment for 48 h before being exposed to a novel conspecific. This procedure mitigates some of the potential anxiogenic effects of the testing environment and allows for the study of social approach selectively, thus allowing us to conclude that the behavioral deficits observed here relate primarily to social motivation, and not to increased general fear produced by a new environment.

Consistent with the well-established ability of ghrelin to increase food intake, elevation of circulating ghrelin either by fasting or by exogenous administration enhances food-seeking behavior in a risky environment as reflected in behavior in the novelty suppressed feeding test^43^. Using this task, we did not find a significant effect of JMV2959, knock-down of GHSR, or rescue on approach to novel food. Given our data, it is tempting to speculate that social behaviors are more sensitive than food-related behavior to the effects of GHSR activation. It is important to note, however, that it is impossible to know whether the food and the novel conspecific can be seen as equally stimulating or rewarding. Furthermore, the singly housed subjects encountered the novel conspecific in a familiar environment, whereas the ad libitum-fed subjects encountered palatable food in a novel environment. This study alone cannot conclude that social behaviors are more sensitive than food-related behaviors to GHSR activation. In addition, given that the VTA underlies a number of reward-seeking behaviors, manipulation of GHSR in the VTA could influence behaviors that include not only social or food seeking behaviors but also behaviors associated with novel stimuli devoid of social or palatable value as demonstrated previously^49^. Notably, disruption in GHSR signaling does not result in exaggerated behavioral coping responses in the face of an axiogenic environment as reflected by the lack of differences in the behaviors recorded on the open field test. Together these data would suggest that GHSR signaling exerts its anxiolytic effect under conditions where the animal has to assess risk in order to reach a goal object. Consistent with this idea, GHSR signaling in the VTA has been shown to increase dopaminergic tone which, in turn, is associated with an increase in motivational state^16^. In the presence of an incentive, either palatable food or a conspecific, an increased motivational state may overcome the anxiety associated with a risky environment. In the absence of such a goal object, however, changes in GHSR signaling may not be observable or may actually be anxiogenic^50^.

Overall, the current findings support a role for GHSR signaling and particularly of GHSR signaling in the VTA in social motivation. These data are consistent with results of previous studies showing that mice lacking GHSR (GHSR^*LoxP/LoxP*^ mice) are more susceptible to the social deficits produced by chronic social stress^5,9^, and that genetic rescue of GHSR in dopamine neurons enhances resilience to chronic social defeat^9^. That the effects of GHSR signaling on social behavior observed in the current studies were independent of exposure to chronic stress or of the acute anxiogenic effects of the testing situation, argues for a more general role of GHSR signaling in social behavior.

The mechanism by which VTA GHSR affects social behavior is unknown but might involve DA, GABA, or glutamate. The VTA contains neurons that produce a number of neurotransmitters including dopamine, GABA and glutamate, and that are involved in behaviors associated with reward and novelty seeking including social exploration^16^. GHSR is expressed in about 50–60% of dopamine neurons and these respond by increasing their firing frequency after direct ghrelin application^18^. The VTA, however, also contains GABA and glutamate secreting neurons and these are also implicated in the regulation of motivated behaviors and positive/negative affective states^51–54^. There is some evidence suggesting that ghrelin also influences GABAergic neurons. For instance, in vivo ghrelin treatment resulted in decreased IPSCs frequency in slice recordings of TTX treated VTA dopamine cells, suggesting an inhibitory effect on GABA synapses^18^. Central ghrelin infusions also seem to increase Fos expression in a subset of GABAergic neurons in the VTA, although it is not clear if this is a direct or indirect effect on these neurons^55^. The effects of ghrelin on VTA glutamate neurons are currently unknown, but ghrelin does increase the activity of VTA glutamatergic synapses onto dopamine neurons as demonstrated by increases in EPSC frequency in recordings from dopamine cells in TTX treated VTA slices following in vivo ghrelin treatment^18^. Further research will determine the relative contribution of these VTA cell groups in relation to GHSR activation.

Social investigation in male rodents allows for identification of potential competitors for resources like food or mates, and also promotes the exchange in cues conveying information about the environment that are known to transfer food preferences^56–59^. Interestingly, male mice given ghrelin injections, or treatments that prevent the breakdown of acyl-ghrelin into its inactive form, result in increased aggression towards other males while JMV2959 decreases aggression^60–62^. Ghrelin also seems to promote social transmission of food preference in rats^56^. In line with these data, our results support the idea that, at least in males, ghrelin receptor signaling, particularly in the VTA, is important for initiating social interactions, and support previous data showing that mice lacking GHSR are more susceptible to become socially defeated. Furthermore, our data support the notion that this vulnerability could be partially reversed by rescuing GHSR in dopamine producing VTA neurons^5,9^. Ultimately, our data support the idea that metabolic hormones can influence behaviors that promote approach towards conspecifics and facilitate the formation of social interactions that could be important for male reproductive behaviors including male/male competition for females and food, and the formation of social hierarchies.

A limitation of the current study is that only male mice were studied and the generalizability of the results to female mice is unclear. The neural circuits underlying motivated behaviors including the motivation to interact with conspecifics are sexually differentiated^63^. This includes the VTA and some of its projections targets like the bed nucleus of the stria terminalis (BNST), medial amygdala, and NAc^63^. In addition, dopamine release into the NAc is greater in females than in males in response to a number of different reinforcers, including the opportunity to socialize with a con-specific^64–67^. The food intake response to ghrelin administration in female mice varies in response to circulating concentration of estradiol such that it is greater when estrogen levels are low^68^. It is not clear, however, how estrogen modulates ghrelin-induced food intake and whether it also modulates the effects of ghrelin on other parameters including social reward and behaviors. This issue is currently being explored in our lab.

One potential confound in assessing the role of GHSR in behavior is the possibility that peripheral or central pharmacological manipulation of GHSR causes malaise that suppresses social or food directed motivation. This is unlikely, however, because previous work showed that peripheral injections of JMV2959 do not produce conditioned taste aversions^69^. Moreover, the JMV2959-treated mice in our study did not show any sudden weight loss that would indicate malaise. Similarly, WT and GHSR KO mice treated with viral vectors did not show any changes in weight or food intake changes that would indicate malaise and an overall decrease in motivation or that would indicate a non-specific effect due to illness.

Data from the current study establish that modulation of the GHSR at the level of the VTA can alter social motivation. However, they do not shed light on how such modulation would typically occur. The evidence for production of ghrelin or some its variants in the brain is controversial^70^ and it is generally accepted that ghrelin enters the brain through a brain transport mechanism that is not yet well characterized^71,72^. Studies in which mapping the entry of labeled ghrelin into the brain suggest that little if any reaches the VTA^71^. In this context it is notable that not all biological functions of the GHSR are dependent on ghrelin binding. The GHSR has high levels of constitutive activity and it can form dimers with other receptors to modulate neuronal activity in a ligand independent manner^73,74^. For example, the GHSR can dimerize with oxytocin receptors, potentially affecting oxytocin receptor signaling and ultimately, modulating social motivation^75^.

The VTA is not the only midbrain structure that expresses the GHSR. These receptors are also localized in areas adjacent to the VTA including the Edinger–Westphal nucleus, the retrorubral field and the substantia nigra, and potentially modulate behavioral responses associated with reward, stress, and motivated states at these sites^20,76^. The Edinger–Westphal nucleus was used as a control site in the GHSR-restoration experiment and it is clear from the data shown in Fig. 4B that GHSR rescue did not extend to this nucleus. Further evidence in support of the specificity of the effects comes from the antagonist study. In this experiment cannulae were aimed towards the anterior portion of the VTA, away from the retrorubral. Moreover, as can be seen in Fig. 3, in which data from mice in which the cannula missed the VTA is shown, there is little evidence for an effect of the antagonist in areas outside the VTA. Nevertheless, there is a need for future studies that examine the role of manipulating GHSR activity in these areas on motivated behaviors more explicitly.

The mesolimbic dopaminergic system has recently emerged as a midbrain hub integrating information associated with social stimuli to generate social motivation and production of social reinforcement^17,77–79^ and the majority of GHSR-expressing cells within the VTA are dopaminergic^18^. However, some GABAergic neurons within this area express c-fos after ghrelin delivery into the brain^55^ suggesting that they too are affected by GHSR activity. Whether GHSR manipulations have their effects on social behavior primarily through dopaminergic or gabaergic neurons requires further study.

Ultimately, the results of this study extended our understanding of the role of GHSR signaling on motivated behaviors, and in particular those associated with social motivation in male mice. This notion is also supported by recent data on male rats treated with ghrelin and tested for social behaviors^80^. Moreover, we showed that modulating GHSR signaling specifically within the VTA is sufficient to affect social motivation. As disrupted social behavior is a common feature of many psychiatric conditions, and since the ghrelin system is sensitive to stress, we suspect that, at least in male individuals, targeting GHSR or using ghrelin analogs could serve as a potential treatment to attenuate social anxiety, a common symptom in many psychiatric conditions including depression, anxiety disorders, and eating disorders.

## Supplementary information

Figure S1

Figure S1 Caption
