# Supplementary figures and images for: Contribution of growth hormone secretagogue receptor (GHSR) signaling in the ventral tegmental area (VTA) to the regulation of social motivation in male mice
Source: Transl Psychiatry. 2021 Apr 20;11:230. doi: 10.1038/s41398-021-01350-6 (PMC8058340; doi:10.1038/s41398-021-01350-6)

Figure S1

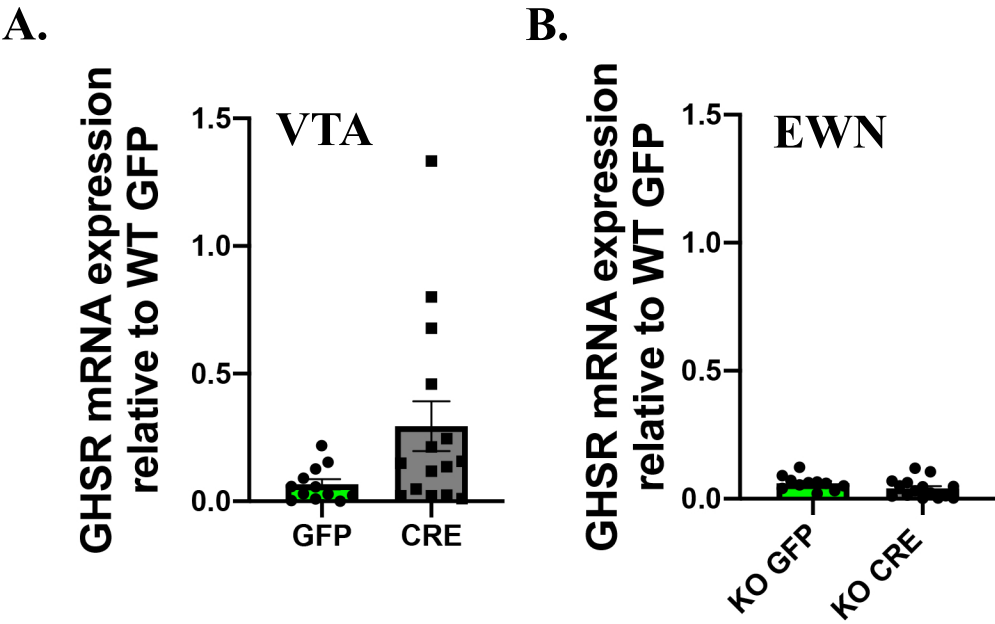

Supplement: Supplementary file 1 — Figure S1 [file 41398_2021_1350_MOESM1_ESM.pdf]
