# Supplementary material for: Contribution of growth hormone secretagogue receptor (GHSR) signaling in the ventral tegmental area (VTA) to the regulation of social motivation in male mice
Source: Transl Psychiatry. 2021 Apr 20;11:230. doi: 10.1038/s41398-021-01350-6 (PMC8058340; doi:10.1038/s41398-021-01350-6)
Supplement: Supplementary file 2 — Figure S1 Caption [file 41398_2021_1350_MOESM2_ESM.docx]

**Figure S1.-**  GHSR expression in GHSR *^LoxP/LoxP^* mice that were infused with a control GFP expressing virus or the pENN.AAV.hSyn.HI.eGFP-Cre.WPRE.SV40 virus to rescue GHSR expression specifically in neurons of the targeted region. As shown in this image, mice infused with the CRE-expressing vector showed increased GHSR mRNA expression in the VTA compared to those receiving the control GFP-expressing vector. Expression of GHSR was variable, however, and only a subset of CRE-transfected mice showed GHSR mRNA expression in the VTA that was higher than 20% of control WT mice. The CRE expressing vector did not increase GHSR expression in the Edinger-Westphal Nucleus (EWN), a region adjacent to the VTA, and one that contains relatively high GHSR expression in WT mice.
